# Gold Nanoparticle-Functionalized Diatom Biosilica as Label-Free Biosensor for Biomolecule Detection

**DOI:** 10.3389/fbioe.2022.894636

**Published:** 2022-05-27

**Authors:** Tongtong Chen, Feifei Wu, Yang Li, Hussein E. Rozan, Xiguang Chen, Chao Feng

**Affiliations:** ^1^ College of Marine Life Science, Ocean University of China, Qingdao, China; ^2^ College of Life Sciences, Qingdao University, Qingdao, China; ^3^ Department of Biochemistry, Faculty of Agriculture, Al-Azhar University, Cairo, Egypt; ^4^ Qingdao National Laboratory for Marine Science and Technology, Qingdao, China

**Keywords:** antibody, biosensor, diatom, biosilica, photoluminescence, gold nanoparticles

## Abstract

Diatom biosilica (DBs) is the cell wall of natural diatom called frustule, which is made of porous hydrogenated amorphous silica possessing periodic micro- to nanoscale features. In this study, a simple, sensitive, and label-free photoluminescence (PL) immune-detection platform based on functionalized diatom frustules was developed. Gold nanoparticles (AuNPs) deposited on poly-dopamine-coated diatom frustules *via in situ* deposition which considerably decreased the intrinsic blue PL intensity of diatom biosilica. Then, goat anti-rabbit immunoglobulin G (IgG) was added to functionalize diatom biosilica-poly-dopamine-AuNPs (DBs-PDA-AuNPs). PL studies revealed that the specific binding with antigen rabbit IgG increased the peak intensity of PL in comparison with the non-complimentary antigen (human IgG). The enhancement in PL intensity of DBs-PDA had a linear correlation with antigen (rabbit IgG) concentration, whose limit of detection (LOD) reached 8 × 10^-6^ mg/ml. Furthermore, PL detection based on DBs-PDA-AuNPs showed a high detection sensitivity with the LOD as low as 8 × 10^-9^ mg/ml and spread over almost eight orders of magnitude, making it suitable for the sensitive quantitative analysis of immune complex compared with traditional fluorescence immunoassay. Hence, the study proves that the AuNP-functionalized diatom frustules can serve as an effective biosensor platform for label-free PL-based immunoassay.

## 1 Introduction

Immuno-detection *via* special recognition with antibody–antigen is an essential tool for clinical detection and diagnosis of diseases rapidly and reliably (Uram et al., 2006; Lin et al., 2013). Compared to electrical and mass measurement methods, optical biosensors have attracted much attention because of their high sensitivity, specificity, and convenience (Dong et al., 2015; Peltomaa et al., 2018; Wang et al., 2019). Label-based optical biosensor such as fluorescence bio-sensing has been widely used in immunoassay, but the photo-bleaching and the limitation of multiplex detection are normally along with the contamination of the sample matrix due to the introduction of labels, which restricts its application in clinic (Gale et al., 2009). Label-free optical bio-sensing platforms can offer real-time monitoring of immune-complex and avoid these drawbacks mentioned earlier (Viji et al., 2014). Surface plasmon resonance (SPR) is one of the most used label-free optical biosensors whose plasmon mode will change when biomolecules bind to the surface of a metal film (Meyer et al., 2011). However, there is a challenge for SPR sensor to detect small biomolecules, such as antibodies, since the binding of small size molecules changes little the refractive index, which reduces the sensitivity of the measurement. On the other hand, the signal uniformity and reproducibility of surface-enhanced Raman spectroscopy (SERS) for small molecules detection, for example, aptamers and antibodies, also confine its uses in the laboratory (Kashif et al., 2020).

Diatoms are mono-cellular eukaryotic phytoplankton widely distributed in oceans and lakes. Diatoms take up soluble silicon to form the cell wall of amorphous silica, which possesses three-dimensional micro- to nano-porous structures (Gordon et al., 2009; De Tommasi et al., 2013). Several studies found that DBs possessed unique photoluminescence (PL) properties which changed upon different biomolecules depositing on its surface, which revealed their promising applications in optical bio-sensing without labeling (Qin et al., 2008; De Tommasi, 2016; LeDuff et al., 2016; Mishra et al., 2020). The PL property of DBs was induced by various surface groups on DBs including Si-OH, Si-H groups, non-bridging oxygen hole centers, and self-trapped excitons (He et al., 2004). Nucleophilic biomolecules such as antibodies enhanced the PL peak intensity by donating electrons to non-radiative defect sites on DBs (Gale et al., 2009; Viji et al., 2014). Within the past few years, DBs has been developed as label-free bio-sensing platforms of PL-based gas sensors for detecting small biomolecules, such as antibodies and explosive derivatives (Viji et al., 2014; Rea et al., 2016; Zhen et al., 2016).

Considering the definite limitations of DBs in immunoassay due to its unreactive nature of the surface, Gregory and his colleagues introduced –NH_2_ on DBs by reaction with 3-aminopropyltrimethoxysilane (APS) and then covalently attached with rabbit immunoglobulin G (IgG) (Gale et al., 2009). The special binding between rabbit IgG with the complimentary antigen goat anti-rabbit IgG increased the PL peak intensity by at least three times in comparison with bare DBs. Furthermore, the enhancement in PL intensity with antigen (goat anti-rabbit IgG) concentration was displayed by a Langmuir model for immune-complex formation. De Stefano et al. (2009) also utilized APTS to modify DBs, using the PL of *Coscinodiscus concinnus* for quantitative analysis and detection of proteins. Zhen et al. (2016) found that the PL emission was partially quenched when TNT binding to the anti-TNT ScFv-functionalized DBs. These results validate that DBs can be used as an optical biosensor platform for PL-based immunoassay. Aforementioned strategies are all based on chemical modification. Poly-dopamine (PDA) can coat the surface of DBs in virtue of its great adhesive properties which is considered an environmentally friendly method for the surface modification of DBs (Uthappa et al., 2019). PDA coating can promote antibody functionalization by virtue of its adhesion properties. In addition, PDA coating can improve the sensitivity of diatoms PL detection by reducing the defect concentration of DBs. Therefore, we used PDA to modify DBs to make it a better optical biosensor based on PL detection.

Noble metal nanoparticles such as gold nanoparticles (AuNPs) and silver nanoparticles (AgNPs) exhibit unique optical properties. They can display the localized surface plasmon resonance (LSPR) phenomenon, whose absorbance will be light very intense when excited at certain wavelengths (Ren et al., 2013; Liu et al., 2018; Petzold and Zollfrank, 2019). According to the surface state luminescence model proposed by Xie et al. (1992), the deposition of metal nanoparticles on the surface of DBs change its surface state and cause its PL intensity to decay. Thus, AuNPs may have the potential to be used in optical biosensors based on PL. In addition, AuNPs possess advantages of being easy to synthesize and modify, good stability, and biocompatibility (Leng et al., 2010). On the other hand, AuNPs possessing ultra-small sizes bound and assemble on the surface of DBs, increasing the available surface area of binding biomolecules and enhancing optical signal sensitivity. Therefore, the deposition of AuNPs on DBs can serve as substrates for optical biosensors.

**SCHEME 1:**
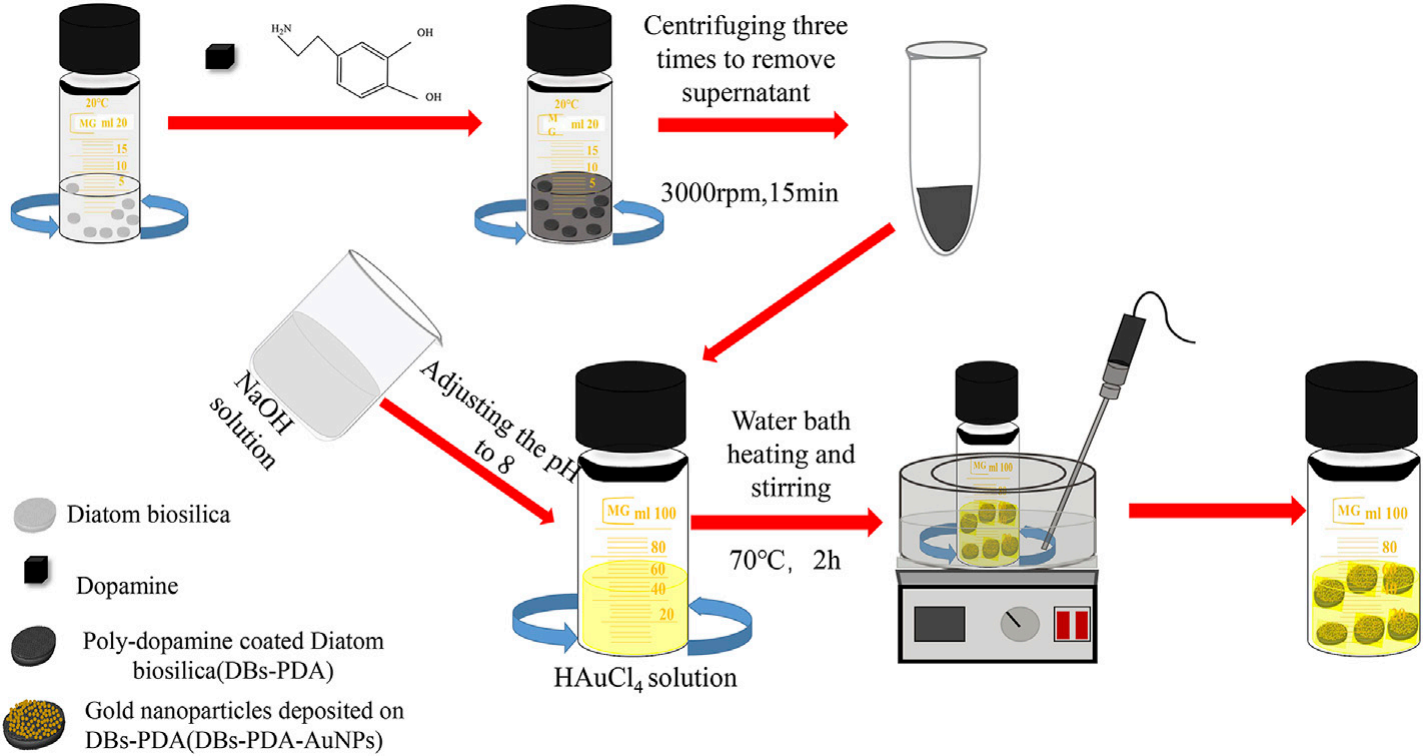
Schematic diagram illustration of the synthesis of DBs-PDA and DBs-PDA-AuNPs.

In this study, we utilized PDA and AuNP-functionalized DBs (Scheme 1). Then, the antibody goat anti-rabbit IgG was attached to PDA coated on DBs (DBs-PDA) and AuNPs deposited on DBs-PDA (DBs-PDA-AuNPs). We examined the correlation of antibody goat anti-rabbit IgG-functionalized DBs-PDA and DBs-PDA-AuNPs with antigen rabbit IgG concentrations in the range from 8 × 10^−9^ to 8 × 10^−2^ mg/ml. DyLight 488-labeled rabbit IgG was employed for immunoassay to compare with the PL and fluorescence detection. Furthermore, in order to validate the universality of DBs as a photo-luminescent immune-sensor, we detected the correlation between the PL peak intensity of rabbit anti-human IgG-functionalized DBs-PDA and DBs-PDA-AuNPs and different concentrations of human IgG (from 8 × 10^−9^ to 8 × 10^−2^ mg/ml). In conclusion, PL detection based on DBs-PDA-AuNPs can be applied to the selectivity, high sensitivity, and label-free immunoassay.

## 2 Materials and Methods

### 2.1 Reagents and Materials

NaOH, bovine serum albumin (BSA), and dopamine hydrochloride (DA) were all purchased from Sigma-Aldrich (United States), and 0.2 M phosphate-buffered saline (PBS) (pH 7.4) was prepared by mixing the stock solutions of KH_2_PO_4_ and K_2_HPO_4_. HAuCl_4_^.^3H_2_O was purchased from Shanghai Aladdin Biochemical Technology Co., Ltd. Rabbit IgG (RIgG), human IgG (HIgG), goat anti-rabbit IgG (GaR), DyLight 488-rabbit IgG (DyLight 488-RIgG), and rabbit anti-human IgG (RaH) were all purchased from Wuhan Boster Biological Technology Ltd. *C. cryptica* (GY-H32) was obtained from the Key Laboratory of Marine Genetics and Breeding, Ocean University of China.

### 2.2 Preparation of Diatom Biosilica

#### 2.2.1 Cultivation of Diatom

*C. cryptica* was cultured in F/2 medium at 20°C with an alternating light/night cycle of 12:12 h for 2 weeks. The diatom cells were obtained *via* filtration and washed three times using deionized water.

#### 2.2.2 Treatment of Diatom Frustules

**FIGURE 1:**
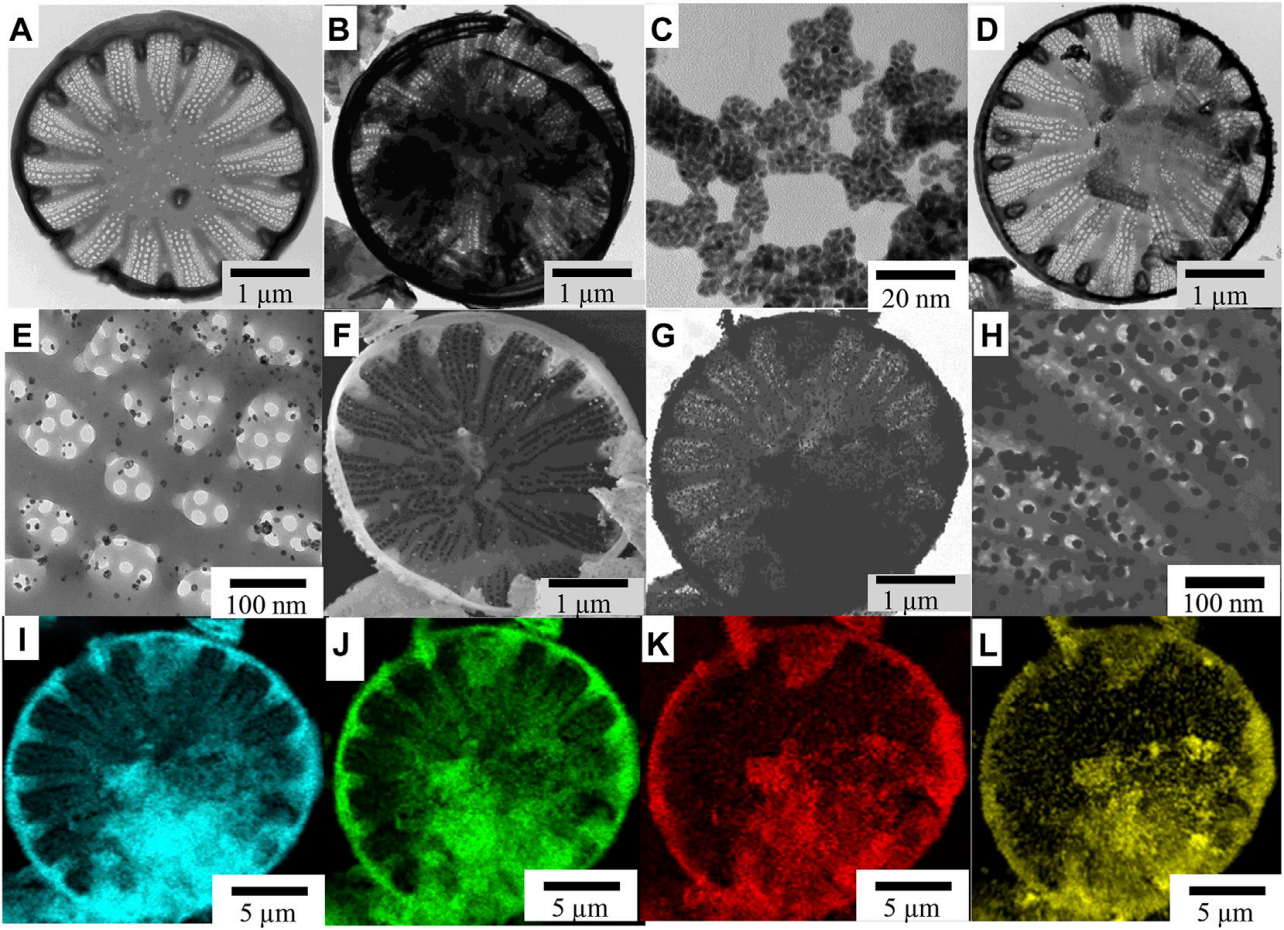
TEM images of **(A)** frustule of C. cryptica, **(B)** PDA-coated diatoms, **(C)** free AuNPs, **(D)** 172 AuNP-deposited DBs-PDA, and **(E)** details of AuNPs on the surface of DBs-PDA. SEM images of 173 **(F)** DBs-PDA-AuNPs, **(G)** DBs-PDA-AuNPs, and **(H)** details of AuNPs on the DBs-PDA. **(I–L)** In total, 174 EDS images of **(I)** Si, **(J)** O, **(K)** C, and (m) Au in DBs-PDA-AuNPs.

*C. cryptica* of 25 g was mixed with 50 ml lye (potassium hydroxide: urea: water = 16:8:66) and stirred evenly, repeated freezing (under−25°C for 5∼6 h each time) and thawing twice frozen twice, and rinsed several times. Then, the pre-cooled treated sample was added to 50 ml of chilled piranha solution (sulfuric acid: hydrogen peroxide = 7:3), which was subsequently heated in a water bath of 75°C for 30 min and stirred constantly, followed by cooling, diluting once, washing by centrifugation several times, and drying in the oven to obtain DBs with the hierarchical porous structure (Figure 1A).

### 2.3 Dopamine Coating on DBs

DBs of 10 mg was dissolved in 10 ml deionized water and stirred continuously for 40 min. Then, 40 mg dopamine hydrochloride (DA) and 200 µl NaOH solution (0.1 M, 50 ml) were added into the diatom solution and stirred evenly. Dopamine hydrochloride (DA) is self-polymerized to poly-dopamine (PDA) in a weak alkaline environment. Then, the sample was centrifuged (3,000 rpm, 15 min) three times, and the supernatant was removed. DBs coated by PDA (DBs-PDA) could be observed using the transmission electron microscope (Figure 1B).

### 2.4 Synthesis of AuNP-Functionalized DBs-PDA

AuNPs were fabricated by the modified method reported previously (Jia et al., 2005). Chloroauric acid (HAuCl_4_^.^3H_2_O) of 85 mg was dispersed in 50 ml ultrapure water and adjusted the pH to 8.0 with 0.1 M NaOH solution. Next, the obtained DBs-PDA was added, heated to 70°C, and continuously stirred for 2 h. Afterward, the resulting solution mixture was centrifuged (3,000 rpm, 15 min) until the absence of Cl^−^ ion and removed the supernatant. Finally, the precipitates were dried completely in the oven.

### 2.5 Preparation of Immuno-Sensors

#### 2.5.1 Immobilization of Antibody on DBs-PDA-AuNPs

DBs functionalized with antibody was prepared by following the methods reported previously (Lai et al., 2013). First, DBs-PDA-AuNP (1.5 mg/ml) suspension was adjusted to the pH 9.0 with 0.1 M K_2_CO_3._ Then, 30 μl goat anti-rabbit IgG was added to 50 μl suspension, by gently mixing at room temperature for 2 h. The sample was then centrifuged for 15 min, followed by mixing with 5% BSA for 1 h for blocking, and washing with PBS twice successively. After centrifugation, antibody-conjugated diatom frustules were collected and re-dissolved in 0.5 ml PBS containing 0.1% BSA. The resulting product was then stored at 4°C for further use.

#### 2.5.2 Immunological Reactions

Stocked antigens were prepared by mixing 10 μl RIgG (with the concentration of 10 mg/ml) with 990 μl PBS. Then, 100 μl DBs-PDA-AuNPs-GaR and RIgG with the concentrations ranging from 8 × 10^−9^ to 8 × 10^−2^ mg/ml were dissolved in a polystyrene 12-well plate containing 2 ml PBS, which was then gently mixed for 2 h at room temperature.

## 3 Results and Discussion

### 3.1 Synthesis and Characterization of DBs-PDA-AuNPs

DBs was isolated from cultured *C. cryptica* using chilled piranha solution to remove the organic matter in cells. The purified DBs was drum-shaped with a diameter of 10∼30 μm and possessed a hierarchical pore structure (Figure 1A). After mixing DBs and DA under weak alkaline conditions (Scheme 1), DA could be oxidized spontaneously, and PDA coating was formed (Figure 1B) *via* the self-polymerization-crosslinking reaction on the surface of DBs. AuNPs were likely to self-assemble with regular size and shape on the surface of DBs-PDA via in situ deposition (Figures 1D, F, G). The free AuNPs were 3∼5 nm diameter and aggregated to form clusters (Figure 1C). In contrast, the AuNPs on DBs-PDA-AuNPs were agglomerated for a larger size with a diameter of 15∼20 nm and distributed evenly on the surface of DBs (Figure 1G). This might be explained by the higher agglomeration tendency of AuNPs when depositing on DBs. EDS images validated that elements of Si (Figure 1I), O (Figure 1J), C (Figure 1K), and Au (Figure 1L) could be found on DBs-PDA-AuNPs which also confirmed the successful immobilization of AuNPs on DBs.

**FIGURE 2:**
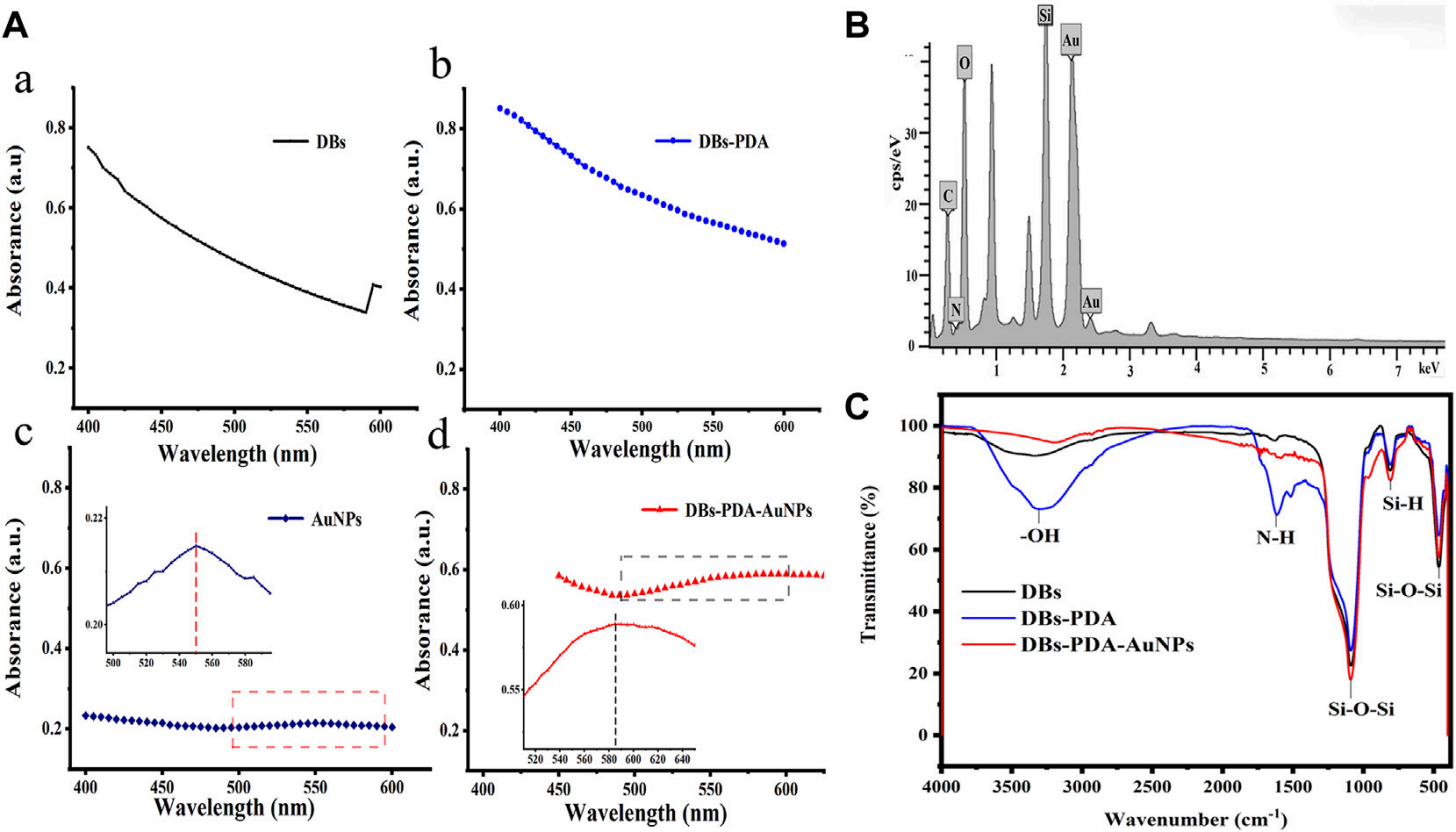
**(A)** UV-Vis absorption spectra of **(a)** DBs, **(b)** DBs-PDA, **(c)** AuNPs (Inset: the UV-Vis absorption spectrum of AuNPs from 495 to 600 nm), and **(d)** DBs-PDA-AuNPs (Inset: the UV-Vis absorption spectrum of DBs-PDA-AuNPs from 510 to 650 nm). **(B)** EDS spectrum of DBs-PDA-AuNPs. **(C)** FITR spectra of DBs, DBs-PDA, and DBs-PDA-AuNPs.

The UV-Vis spectra of DBs, DBs-PDA, AuNPs, and DBs-PDA-AuNPs showed that there were no characteristic absorption peaks in the spectra of DBs and DBs-PDA at the wavelength range of 400–600 nm, and their spectral shapes were very similar (Figure 2A,B). The noticeable characteristic absorption band peaking at 550 nm, which was an indicative of the formation of AuNPs, was observed in the UV-Vis spectrum (Figure 2C). After AuNPs deposited on the surface of DBs-PDA, the absorption peak intensity of DBs-PDA-AuNPs increased in comparison with AuNPs alone. The absorption band of DBs-PDA-AuNPs was red-shifted and the peak appeared at about 580 nm, indicating the aggregation of AuNPs on DBs-PDA (Figure 2d), which was consistent with the agglomeration tendency observed in the SEM image (Figure 1H). The energy dispersive X-ray spectrum (EDS) of DBs-PDA-AuNPs presented the distribution of elements including C, O, Si, and Au and further validated the existence of AuNPs (Figure 2B). The FT-IR spectra clearly showed characteristic peaks for DBs, including Si–O–Si bending at 466 and 806 cm^−1^, Si–O–Si stretching at 1,090 cm^−1^, and O–H stretching of surface-bound hydroxyl groups at 3,147 cm^−1^, which mainly included bound water, H–O–Si, and pyrocatechol of poly-dopamine. After PDA coated on DBs, the strong peaks at 1,615 cm^−1^ shown in the spectrum corresponded to the typical absorption peak of amide bonds on the PDA chain (Yang et al., 2014). However, when AuNPs deposited on DBs-PDA, the amide bonds almost disappeared, which might suggest the formation of Au–N. According to the infrared spectra, the characteristic absorption peak at −3,147 cm^−1^ weakened after AuNPs functionalized DBs, suggesting that the free Si–OH on DBs reacted with AuNPs to form the Si–O–Au group (Figure 2C). From the aforementioned results, it could be concluded that AuNPs had been successfully fixed on the surface of DBs-PDA.

**FIGURE 3:**
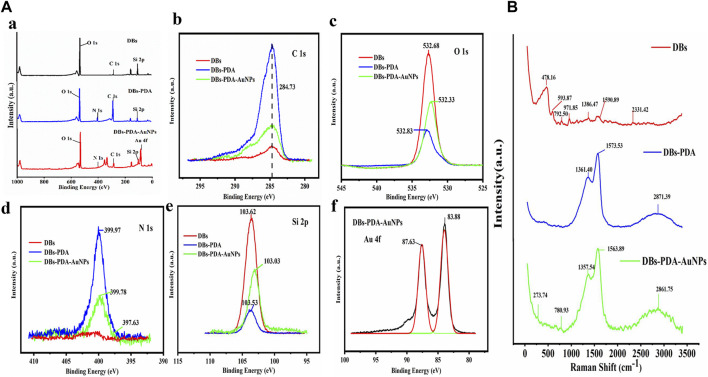
**(A)** XPS spectra of DBs, DBs-PDA, and DBs-PDA-AuNPs. **(a)** XPS survey spectrum of DBs and PDA-coated DBs as well as AuNP-deposited DBs-PDA. **(b)** C_1s_ scan spectrum, **(c)** O_1s_ scan spectrum, **(d)** N_1s_ scan spectrum, **(e)** Si_2p_ scan spectrum, and **(f)** Au_4f_ scan spectrum. **(B)** SERS spectrum of DBs, DBs-PDA, and DBs-PDA-AuNPs.

The XPS survey spectrum indicated that AuNPs were successfully deposited on the surface of DBs (Figure 3A). The functionalization of PDA coating and AuNPs only displays a peak C1s at 284.73 eV (Figure 3B). After AuNPs deposited on DBs, the two components of Au 4f_7/2_ were at binding energy = 83.9 and 87.6 eV, which indicated the emergence of Au^0^ and the ion of Au^+1^ on the surface of DBs-PDA-AuNPs, respectively. This result demonstrated that the chloroauric acid was reduced to AuNPs and successfully immobilized on DBs-PDA (Figure 3f). The appearance of the N_1s_ peak of DBs-PDA and DBs-PDA-AuNPs near 399 eV could be attributed to the existence of NSi_2_O (Figure 3d). The Si_2p_ of DBs located at 103.62 eV was attributed to SiO_2_, and the peak value of DBs-PDA was shifted toward a lower binding energy around 0.09 eV (from 103.6 to 103.5 eV), implying that part of the Si–OH bond had been transferred into Si–N bond. In addition, the Si_2p_ of DBs-PDA-AuNPs located at 103.0 eV was shifted to lower binding energy by 0.5 eV than that of DBs-PDA, suggesting that part of the Si–N bond had changed to Si–Au bond (Figure 3e). Thus, AuNPs were more likely to form Si–O–Au bonds with the free Si–OH on the surface of DBs.

**FIGURE 4:**
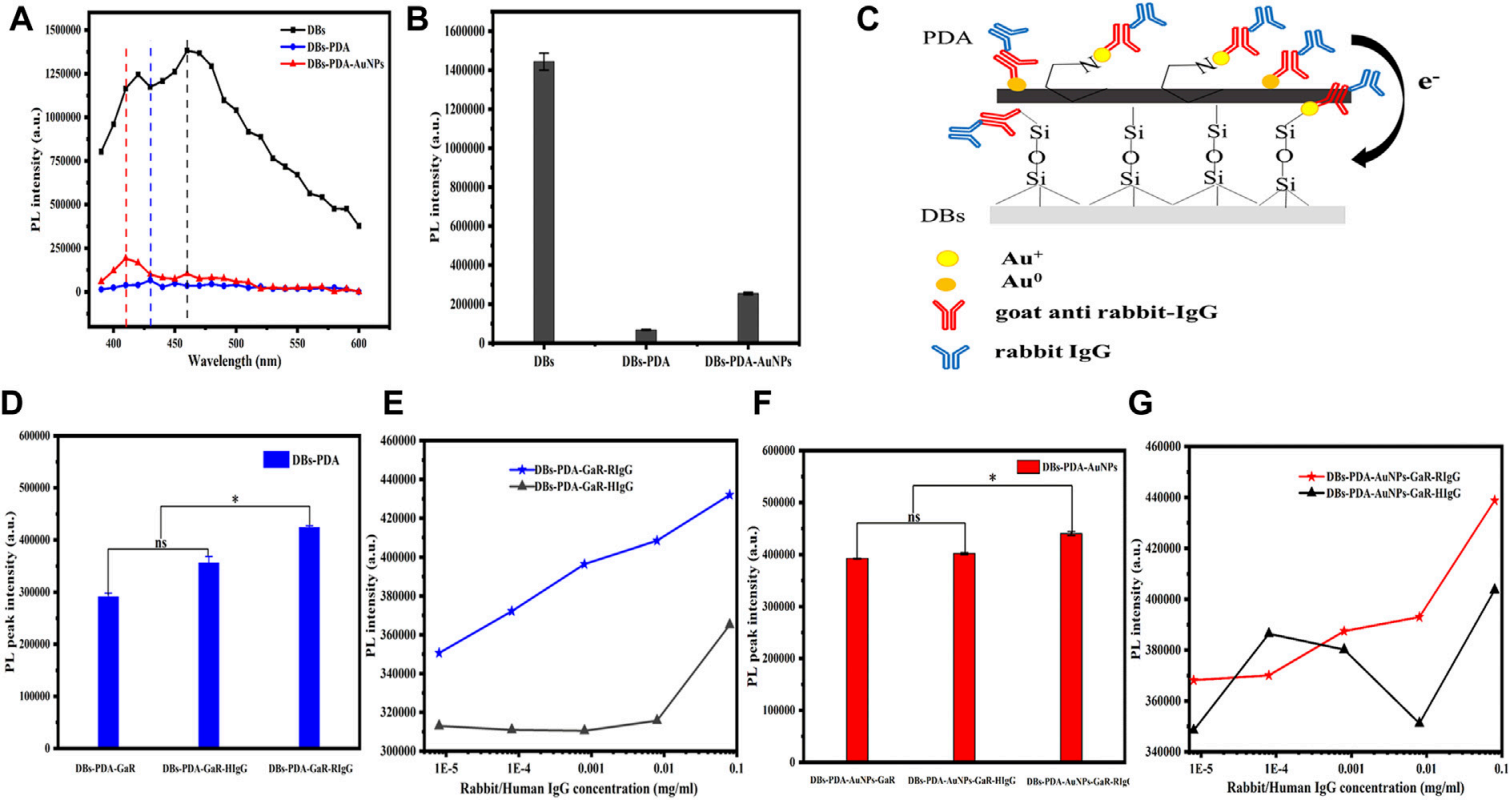
**(A)** PL spectra of DBs, DBs-PDA, and DBs-PDA-AuNPs. **(B)** Comparison of PL peak intensity of DBs, DBs-PDA, and DBs-PDA-AuNPs. **(C)** Schema of functionalized DBs-PDA-AuNPs with antibody GaR and complimentary antigen RIgG. **(D)** Comparison of the PL peak intensity of DBs-PDA-GaR (DBs-PDA-antibody), DBs-PDA-GaR with RIgG (DBs-PDA-antibody-comp. antigen), and DBs-PDA-GaR with HIgG (DBs-PDA-antibody-non-comp. antigen). **(E)** Comparison of PL peak intensity variation of DBs-PDA-GaR versus RIgG and HIgG (from 8 × 10^−6^ to 8 × 10^−2^ mg/ml), respectively. ns represents no significant difference, *p* > 0.05* represents a significant difference, and *p* < 0.05. **(F)** Comparison of PL peak intensity of DBs-PDA-AuNPs-GaR, DBs-PDA-AuNPs-GaR with RIgG, and DBs-PDA-AuNPs-GaR with HIgG. **(G)** Comparison of PL peak intensity variation of DBs-PDA-AuNPs-GaR versus RIgG and HIgG (from 8 × 10^−6^ to 8 × 10^−2^ mg/ml), respectively. Excitation wavelength was 360 nm.

The Raman spectrum of DBs had a characteristic peak at 478.2 cm^−1^, corresponding to Si–O–Si stretching vibration (red curve). Due to the introduction of PDA, peaks of −1,362 and −1,571 cm^−1^ of DBs-PDA and DBs-PDA-AuNPs both appeared in the Raman spectra which were attributed to the presence of aromatic phenyl in PDA (blue and green curves). The characteristic absorption peak at 2,850–2,923 cm^−1^ was attributed to the stretching vibration of -CH. DBs-PDA-AuNPs (green curve) had a weak characteristic peak at −270 cm^−1^ which did not appear in the Raman spectra of DBs and DBs-PDA (red and blue curve) (Figure 3b). It was supposed that this characteristic absorption peak was concerned with the formation of N–Au (Figure 4C). The aforementioned results showed that when AuNPs were deposited on the surface of DBs-PDA, PDA chelated Au (I) *via* catechol and amino groups. Therefore, AuNPs were immobilized on the surface of DBs-PDA.

### 3.2 PL Studies

#### 3.2.1 PL Detection of DBs-PDA-AuNPs

The PL properties of DBs, DBs-PDA, and DBs-PDA-AuNPs were evaluated using the multimode microplate reader. Several studies have demonstrated that DBs have intrinsic blue photoluminescence due to the surface defects on the DBs, (Rorrer et al., 2007; Arteaga-Larios et al., 2014; De Tommasi, 2016) which was also confirmed by our studies. It was observed that bare DBs emitted strong blue photoluminescence centered at 450 nm when samples were excited at 360 nm (Figure 4A). After being coated by PDA, the PL intensity of DBs-PDA was violently quenched, which displayed 13-fold lower than that of bare DBs (Figure 4B). The peak wavelength for DBs-PDA exhibited a blue shift from 450 to 430 nm caused by the interaction of PDA and the surface of DBs (Figure 4A). Defect concentration affected the PL properties of DBs. Thus, the decreased PL peak intensity and blue shift of DBs-PDA might be ascribed to the reduction of defect concentration when there is existence of PDA coating on the surface of DBs. Fedorenko et al. (2020) reported the similar results that PDA coating on ZnO nanorods caused the decrease of the defect concentration and the PL peak intensity. The PL emission spectra clearly showed 6-fold weaker PL peak intensity of DBs-PDA-AuNPs than that of bare DBs (Figure 4B). AuNPs functionalized DBs by forming Si–O–Au bonds, which induced the emergence of a new non-radiative center on the surface of DBs and decreased the PL peak intensity of DBs-PDA-AuNPs. The PL spectrum of DBs-PDA-AuNPs showed an obvious blue shift (from 450 to 410 nm). This result highlighted that AuNPs deposited on DBs *via in situ* polymerization would lead to the blue shift of the peak position. However, the PL peak intensity of DBs-PDA-AuNPs was one-fold higher than that of DBs-PDA. It was supposed that Au^+^, which was formed by Au^3+^ reduction, combined with amino functional groups of PDA on the surface of DBs-PDA-AuNPs to form N–Au bonds. The interaction between AuNPs and PDA induced the enhancement of surface passivation on DBs-PDA-AuNPs, which affected the surface recombination centers, the radiative points responsible for the PL of DBs. This inference could explain the rise of PL peak intensity after AuNPs functionalized DBs-PDA compared with DBs-PDA alone (Figure 4C).

#### 3.2.2 Specificity

Our experiments validated the specificity of antibody-functionalized DBs-PDA-AuNPs with its complimentary antigen. First, when GaR-functionalized DBs-PDA (DBs-PDA-GaR) bonds with non-complimentary antigen HIgG, the PL peak intensity of DBs-PDA-GaR-HIgG showed no significant change. However, when the complimentary antigen RIgG bound with DBs-PDA-GaR, the PL peak intensity was significantly different (*p* < 0.05) which increased by more than 45% than that of DBs-PDA-GaR alone. (Figure 4D). The PL peak intensity of the formed complex between RIgG and GaR-functionalized DBs-PDA-AuNPs (DBs-PDA-AuNPs-GaR) changed significantly (*p* < 0.05) which increased more than 12% than DBs-PDA-AuNPs-GaR. However, no significant change in the PL peak intensity was observed after DBs-PDA-AuNPs-GaR functionalized with non-complimentary antigen HIgG (Figure 4F). The reason behind the peak PL intensity increase of the immune complex was the forming of nucleophilic molecules on the surface of DBs that transferred electrons to non-radiative defect sites on DBs (Figure 4C). In immune complex formation, the PL intensity of antibody binding on DBs-PDA-GaR and DBs-PDA-AuNPs-GaR showed a linear increase with the antigen RIgG concentration ranging from 8 × 10^−6^ to 8 × 10^−2^ mg/ml whereas non-complimentary antigen HIgG did not (Figures 4E,G). These results illustrated the specificity of DBs-PDA and DBs-PDA-AuNPs applied to immune-detection.

### 3.4 RIgG Detection Based on DBs-PDA-AuNPs

**FIGURE 5:**
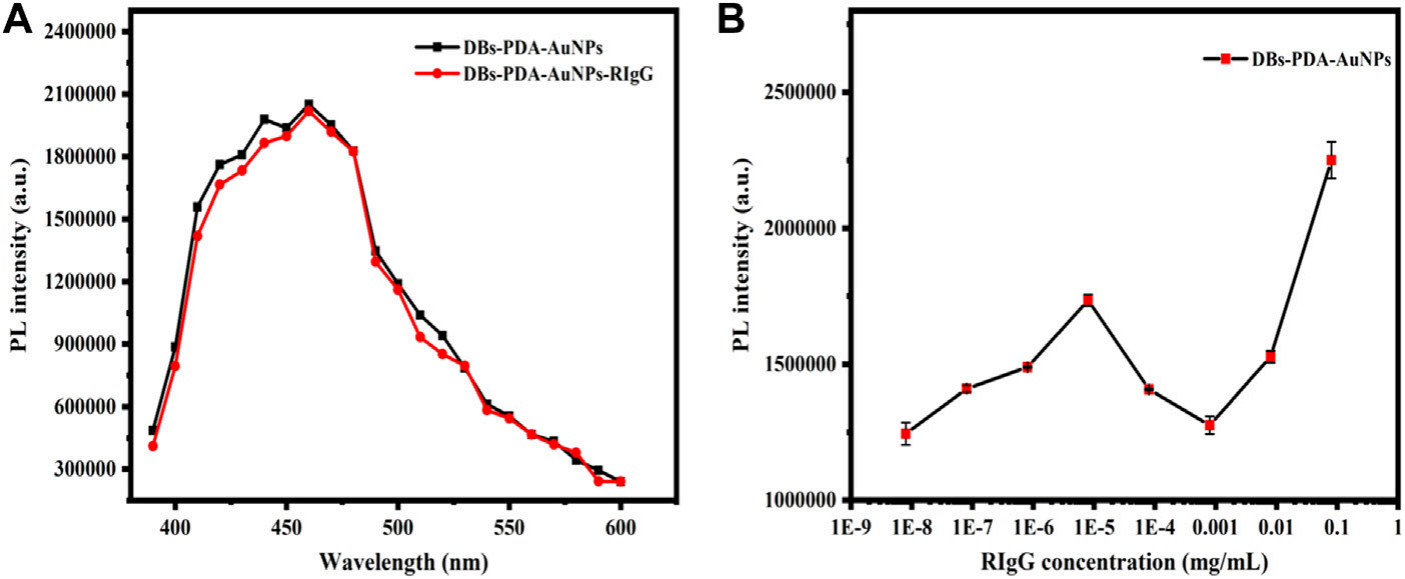
**(A)** PL spectra of DBs-PDA-AuNPs and DBs-PDA-AuNPs-RIgG. **(B)** PL spectra showed DBs-PDA-AuNPs binding to different concentrations of RIgG (8 × 10^−9^∼8 × 10^−2^ mg/ml). Excitation wavelength was 360 nm.

**FIGURE 6:**
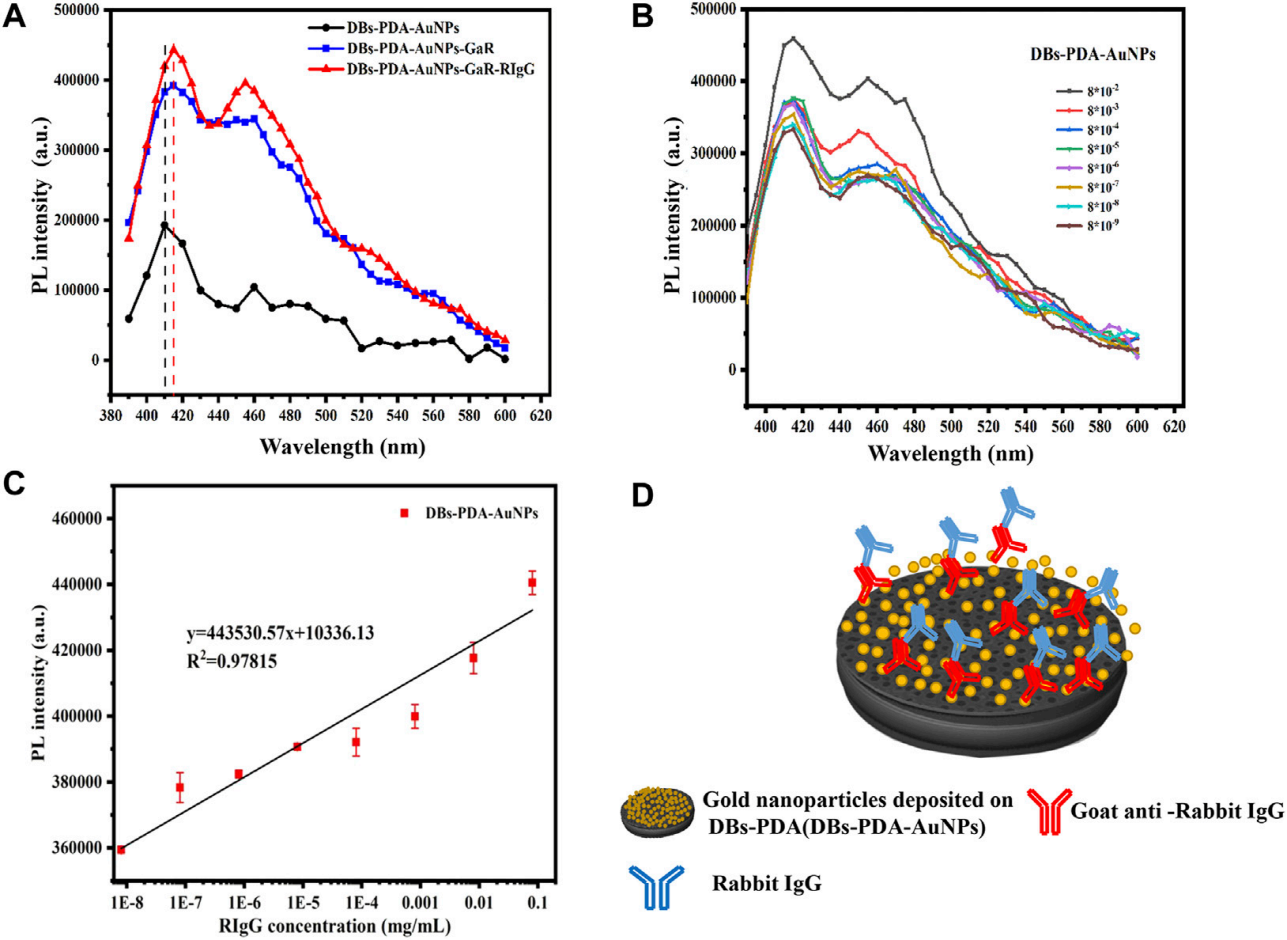
**(A)** PL spectra of DBs-PDA-AuNPs, DBs-PDA-AuNPs-GaR, and DBs-PDA-AuNPs-GaR with its complimentary antigen (RIgG). **(B)** PL spectra showed DBs-PDA-AuNPs-GaR binding to different concentrations of RIgG (from 8 × 10^−9^ to 8 × 10^−2^ mg/ml). **(C)** PL intensity of DBs-PDA-AuNPs-GaR labeled with different concentrations of RIgG (from 8 × 10^−9^ to 8 × 10^−2^ mg/ml) with a coefficient of determination (*R* ^*2*^) of 0.97815. Excitation wavelength was 360 nm. **(D)** Schema of RIgG binding with DBs-PDA-AuNPs-GaR.

According to PL spectra of DBs-PDA-AuNPs and DBs-PDA-AuNPs-RIgG, when RIgG functionalized DBs-PDA-AuNPs, its peak PL intensity basically did not change compared with DBs-PDA-AuNPs, and the characteristic absorption peak did not change either (Figure 5A). As the concentration of RIgG changed (from 8 × 10−^9^ to 8 × 10^−2^ mg/ml), the peak PL intensity of DBs-PDA-AuNPs-RIgG did not show a linear relationship with the concentration change (Figure 5B). These results indicated that RIgG did not bind to the surface of DBs through reaction with AuNPs. A minute quantity of RIgG deposited on DBs, which would not affect the PL property of DBs-PDA-AuNPs. However, after GaR functionalized DBs-PDA-AuNPs, the peak PL intensity was 2-fold as that of DBs-PDA-AuNPs. In addition, the enhancement of defect concentration on the surface of DBs-PDA-AuNPs-GaR contributed to a slight blue shift of the PL characteristic peak (from 410 to 415 nm). Furthermore, the specific binding between GaR and its complimentary antigen RIgG induced the PL intensity, with a 10% increase in comparison with DBs-PDA-AuNPs-GaR, and more than one-fold higher than that of DBs-PDA-AuNPs (Figure 6A). These results confirmed that the nucleophilic complex attaching to DBs could increase PL intensity. The PL emission spectra of DBs-PDA-AuNPs showed that the peak PL intensity was linearly increased with RIgG concentrations in the range of 8 × 10^−9^ to 8 × 10^−2^ mg/ml without any peak shifts (Figure 6B). The increase in PL peak intensity with various RIgG concentrations was described by the best-fit equation: *y* = 443530.57x + 1.03E04 and the coefficients of determination (*R* ^*2*^) of 0.9781 (Figure 6C). These results illustrated that antigen RIgG molecules were adsorbed on the surface of DBs-PDA-AuNPs-GaR and involved in the formation of immuno-complex (Figure 6D). As a platform of immuno-detection, DBs-PDA-AuNPs showed an outstanding PL performance for detecting RIgG with the LOD reaching as low as 8 × 10^−9^ mg/ml.

**FIGURE 7:**
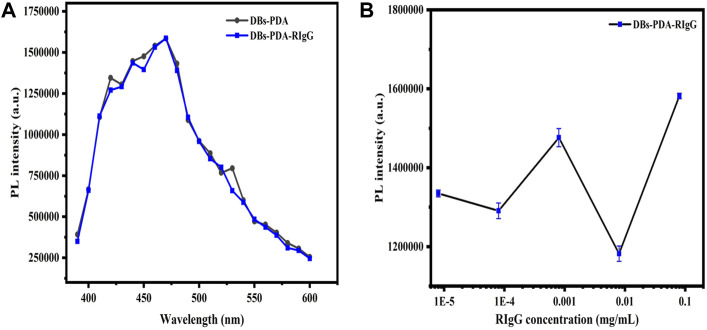
**(A)** PL spectra of DBs-PDA and DBs-PDA-RIgG. **(B)** PL spectra showed DBs-PDA binding to different concentrations of RIgG (from 8 × 10^−9^ to 8 × 10^−2^ mg/ml). Excitation wavelength was 360 nm.

**FIGURE 8:**
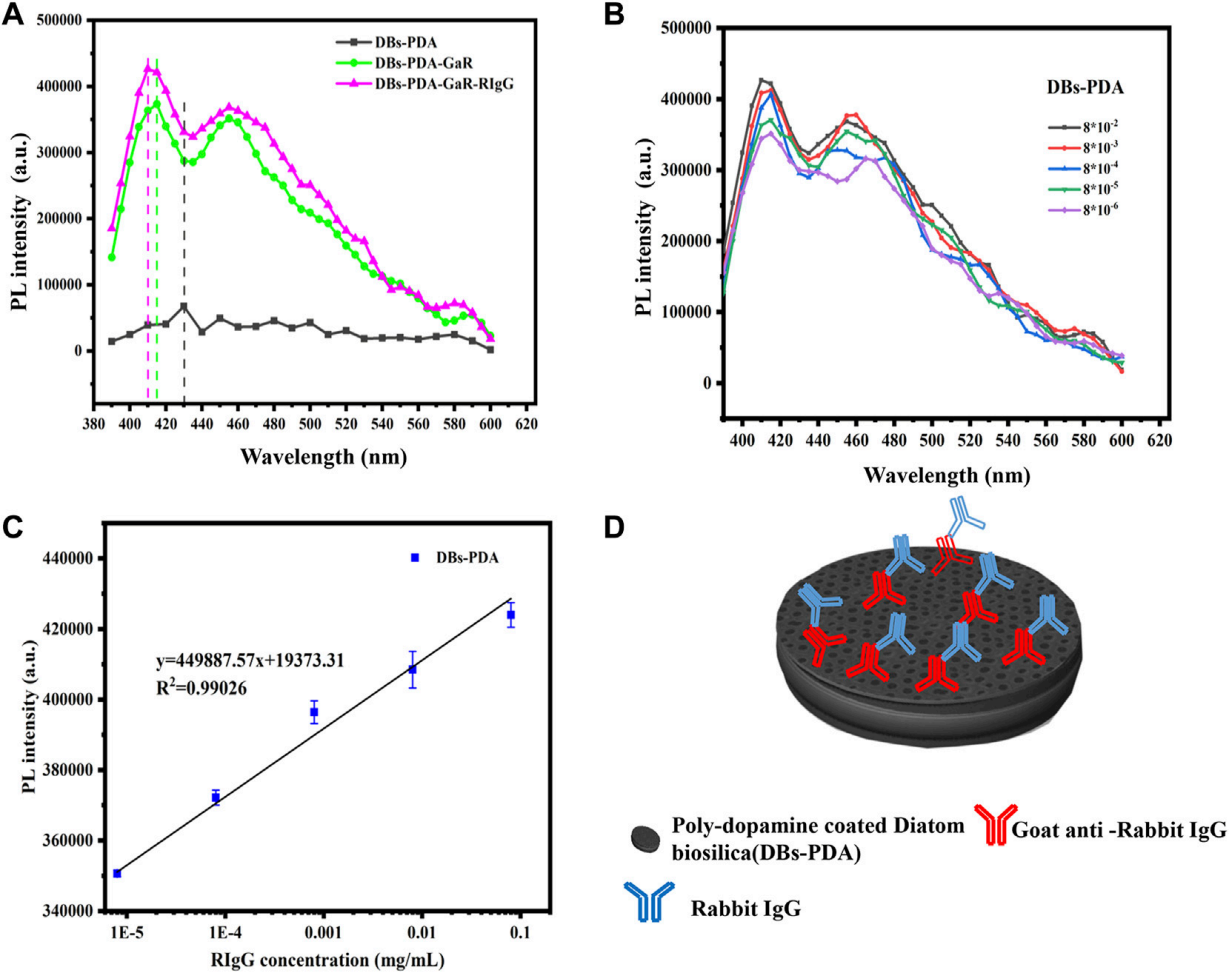
**(A)** PL spectra of DBs-PDA, DBs-PDA-GaR, and DBs-PDA-GaR against its complimentary antigen (RIgG). **(B)** PL spectra showed DBs-PDA-GaR versus different concentrations of RIgG (from 8 × 10^−6^ to 8 × 10^−2^ mg/ml). **(C)** PL intensity of DBs-PDA-GaR labeled with different concentrations of RIgG (from 8 × 10^−9^ to 8 × 10^−2^ mg/ml) with a coefficient of determination (*R* ^*2*^) of 0.97815. Excitation wavelength was 360 nm. **(D)** Schema of RIgG binding with DBs-PDA-GaR.

For DBs-PDA groups, the PL emission spectra clearly indicated that the PL peak intensity of RIgG-functionalized DBs-PDA (DBs-PDA-RIgG) barely changed and without any peak shift as compared to that of DBs-PDA alone (Figure 7A). Furthermore, there was no linear relationship between the PL peak intensity and RIgG concentrations (from 8 × 10^−9^ to 8 × 10^−2^ mg/ml) (Figure 7B). This indicated that RIgG did not react with the free groups on the surface of DBs-PDA, and only a small amount of RIgG was deposited on DBs-PDA. Thus, the PL performance of DBs-PDA-RIgG was consistent with that before RIgG deposition. When RIgG bonds with DBs-PDA-GaR (Figure 8D), the PL peak intensity was enhanced by more than 10%, and a slight blue peak shift could be observed in PL emission spectra which was due to the decrease of defect concentration on the surface of DBs-PDA (Figure 8A). In immune-complex formation, the PL peak intensity showed a linear increase with concentrations of the complementary antigen RIgG from the range of 8 × 10^−6^ to 8 × 10^−2^ mg/ml (Figure 8B). The PL emission spectra clearly showed the increase in PL peak intensity with various RIgG concentrations (from 8 × 10^−9^ to 8 × 10^−2^ mg/ml). However, the peak PL intensity of DBs-PDA was found to be increasing linearly with the RIgG concentrations from 8 × 10^−6^ to 8 × 10^−2^ mg/ml. The regression equation was y = 19373.31x + 4.49E05 with the correlation coefficient of determination (*R* ^*2*^) of 0.99026 (Figure 8C). The limit of PL detection for RIgG based on DBs-PDA reached 8 × 10^−6^ mg/ml, which was three orders of magnitude lower than that of DBs-PDA-AuNP-based detection.

### 3.5 PL-Detection Compared With Fluorescence Immunoassay

**FIGURE 9:**
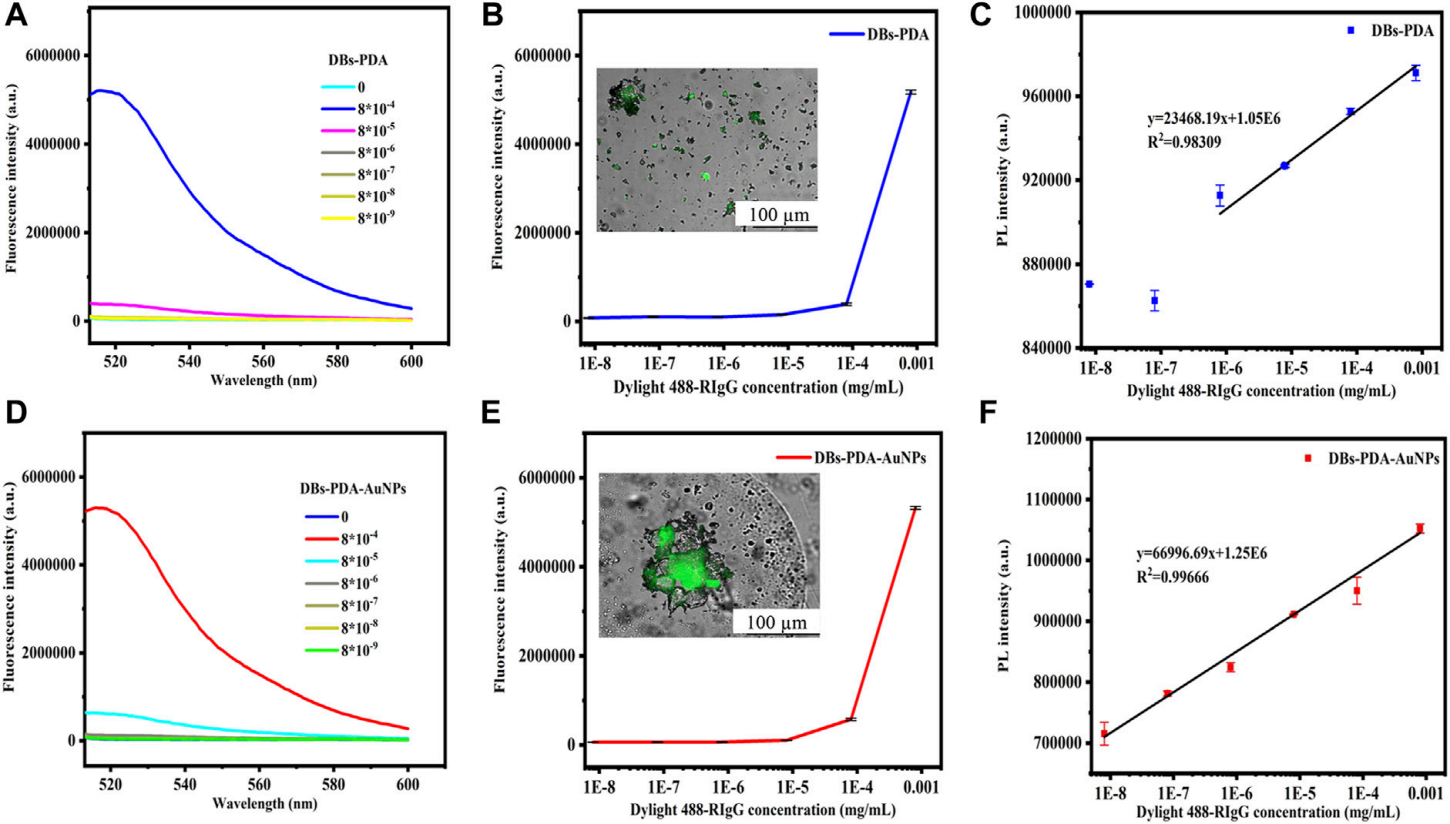
Fluorescence spectra of GaR-functionalized **(A)** DBs-PDA and **(D)** DBs-PDA-AuNPs challenged with DyLight 488-RIgG with concentrations from 8 × 10^−9^ to 8 × 10^−4^ mg/ml. Peak fluorescence intensity variations of GaR-functionalized **(B)** DBs-PDA and **(E)** DBs-PDA-AuNPs versus DayLight 488-RIgG with different concentrations (from 8 × 10^−9^ to 8 × 10^−4^ mg/ml). Inset: fluorescence images of **(B)** GaR-functionalized DBs-PDA and **(E)** DBs-PDA-AuNPs challenged with DyLight 488-RIgG, respectively. Excitation wavelength was 488 nm. PL emission spectra of goat-anti-RIgG-functionalized **(C)** DBs-PDA and **(F)** DBs-PDA-AuNPs binding to different concentrations of DyLight 488-RIgG (from 8 × 10^−9^ to 8 × 10^−4^ mg/ml) with the coefficient of determination (*R* ^*2*^) of 0.98309 and 0.99666, respectively. Excitation wavelength was 360 nm.

Several studies have demonstrated the potential of the AuNP-based fluorescence-labeling probe technique applied on immunoassay and biosensors. The fluorescence spectra showed that GaR functionalized DBs-PDA and DBs-PDA-AuNPs and challenged with DyLight 488-RIgG concentrations ranging from 8 × 10^−9^ to 8 × 10^−4^ mg/ml. The fluorescence peak intensity was not proportional to the change of antigen DyLight 488-RIgG concentrations (Figures 9A,D). In addition, after binding with different DyLight 488-RIgG concentrations ranging from 8 × 10^−9^ to 8 × 10^−4^ mg/ml, the fluorescence peak intensity was observed in fluorescence spectra. Two curves showed a similar variation trend and further indicated that there was no linear relation between fluorescence peak intensity and DyLight 488-RIgG concentrations. When challenged with RIgG-labeled DyLight 488, the entire surface of DBs-PDA and DBs-PDA-AuNPs both emitted green light which illustrated the successful binding between DyLight 488-RIgG and GaR-functionalized DBs-PDA and DBs-PDA-AuNPs, respectively (Figures 9B,E, Inset). With the increase of DyLight 488-RIgG concentrations from 8 × 10^−9^ to 8 × 10^−4^ mg/ml, the PL peak intensity of DBs-PDA was found increasing linearly over a range from 8 × 10^−9^ to 8 × 10^−4^ mg/ml. The regression equation was y = 23468.19x + 1.05E06 with the coefficient of determination (*R* ^*2*^) of 0.98309 (Figure 9C). The correlation of DBs-PDA-AuNPs-GaR versus different concentrations of antigen DyLight 488-RIgG could be found in the PL spectrum. The peak intensity of PL was observed increasing linearly with RIgG concentrations over a range from 8 × 10^−9^ to 8 × 10^−4^ mg/ml. The regression equation was y = 66996.69x + 1.25E06 with the coefficient of determination (*R* ^*2*^) of 0.99666 (Figure 9F). The limit of PL detection for DyLight 488-RIgG based on DBs-PDA-AuNPs was three orders of magnitude higher than that based on DBs-PDA. Furthermore, the PL detection was more suitable in selective and quantitative detection of antigen DyLight 488-RIgG than fluorescence immunoassay.

### 3.6 HIgG Detection Based on DBs-PDA-AuNPs

**FIGURE 10:**
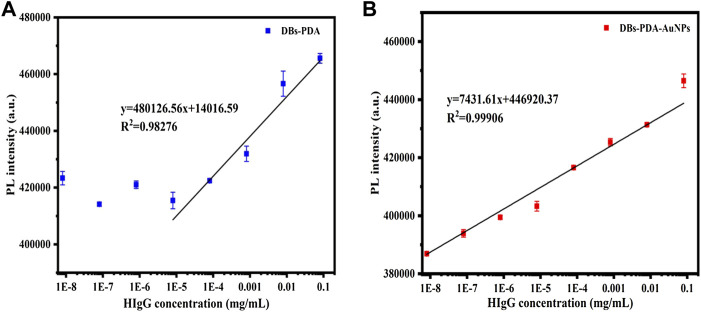
PL peak intensity variations of **(A)** DBs-PDA and **(B)** DBs-PDA-AuNPs versus the HIgG concentration with a coefficient of determination (*R* ^*2*^) of 0.98276 and 0.99906, respectively. Excitation wavelength was 360 nm.

The PL detection for HIgG was conducted to validate the universality of DBs-PDA and DBs-PDA-AuNPs as label-free optical biosensors for immune-detection. The increase in PL peak intensity of RaH-functionalized DBs-PDA-AuNPs (DBs-PDA-AuNPs-RaH) with antigen HIgG concentrations ranged from 8 × 10^−9^ to 8 × 10^−2^ mg/ml was displayed. The PL peak intensity was found increasing linearly over a wide range from 8 × 10^−9^ to 8 × 10^−2^ mg/ml. The regression equation was y = 7,431.61x + 4.47E05 with the correlation coefficient of determination (*R* ^*2*^) of 0.99906 (Figure 10B). The LOD of DBs-PDA-AuNPs could reach 8 × 10^−9^ mg/ml. With the increase of HIgG concentrations, the PL peak intensity was linearly related to the concentration of HIgG concentrations from 8 × 10^−6^ to 8 × 10^−2^ mg/ml with the regression equation of *y* = 480126.56x + 1.40E04 and the correlation coefficient (*R* ^*2*^) of 0.98276 (Figure 10A). The LOD for HIgG detection based on DBs-PDA was three orders of magnitude lower than DBs-PDA-AuNPs. This result suggested that DBs-PDA-AuNPs were more suitable for quantitative detection of HIgG concentrations. Furthermore, PL detection based on DBs-PDA-AuNPs had the universality to be applied for immune detection in clinical samples.

## 4 Conclusion

In this present work, DBs obtained from the centric diatom *C. cryptica* were employed to establish a nanostructured immune assay platform coated with PDA and functionalized *via* AuNPs, which induced the decrease of defect concentration and quenched the PL peak intensity of DBs. N–Au formed after AuNPs reacted with PDA and further enhanced the surface passivation of DBs, which amplified the PL peak intensity of DBs-PDA-AuNPs as compared to DBs-PDA. The PL detection for RIgG based on DBs-PDA and DBs-PDA-AuNPs all exhibited high biological specificity. Nucleophilic complex formation increased the PL peak intensity of DBs-PDA and DBs-PDA-AuNPs. Furthermore, physisorption between antibody and DBs-PDA-AuNPs was likely to concentrate plenty of complimentary antigens on the surface, which resulted in high sensitivity and a decrease in the LOD. The LOD of PL detection for RIgG and HIgG based on DBs-PDA-AuNPs could reach 8 × 10^−9^ mg/ml, which was three orders of magnitude higher than that of DBs-PDA. In addition, PL detection for DyLight 488-RIgG based on DBs-PDA-AuNPs exhibited higher LOD than fluorescence immunoassay. As such, antibody-functionalized DBs-PDA-AuNPs could serve as an efficient optical biosensor platform for the selective, sensitive, and label-free PL-based immunoassay.

## Data Availability

The original contributions presented in the study are included in the article/Supplementary Material; further inquiries can be directed to the corresponding author.
